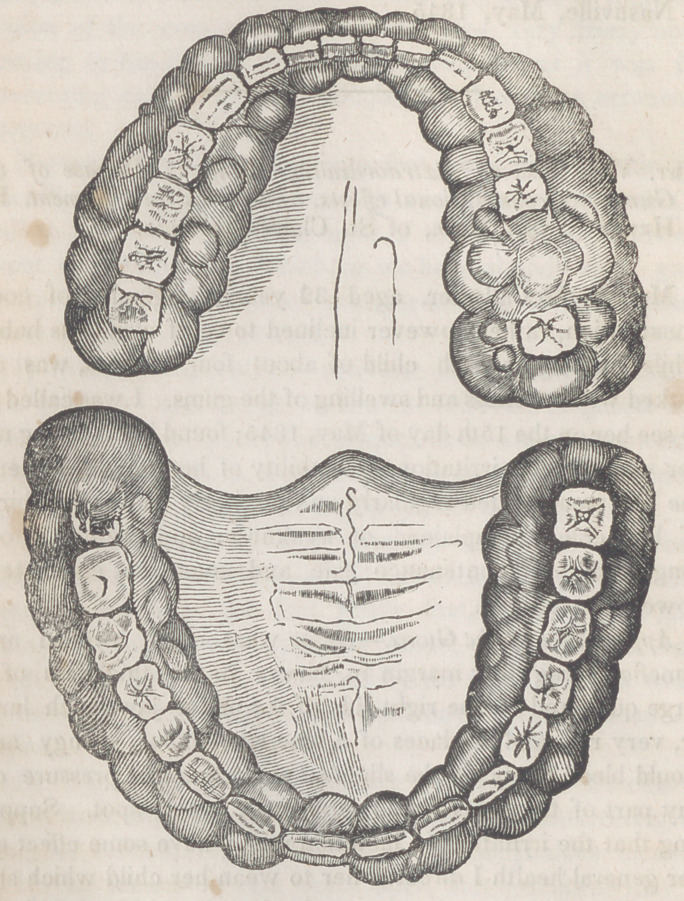# Case of Extraordinary Fungous Disease of the Gums, Its Constitutional Effects, and Successful Treatment

**Published:** 1845-11

**Authors:** Henry West

**Affiliations:** St. Clairsville, Ohio


Art. V.—*Case of Extraordinary Fungous Disease of the Gums, its constitutional effects, and successful treatment.* By Henry West, M.D., of St. Clairsville, Ohio.

Mrs. B. McCallester, aged 32 years, originally of good constitution, rather however inclined to be of strumous habit, whilst nursing her fifth child of about four months, was attacked with soreness and swelling of the gums. I was called in to see her on the 15th day of May, 1845; found her laboring under considerable irritation and debility of her general system; she had mensturated regularly and profusely since the birth of her child, complained of weakness; appetite not good; tongue coated; countenance pale and somewhat emaciated; bowels constipated.

*Appearance of the Gums.*—Gums were much swelled and tumefied along their margin in places to the thickness of a large quill, and on the right side of the under jaw much larger, very red, and in places of a blue black color, spongy and would bleed freely on the slightest scratch; slight pressure on any part of the gums would produce a black spot. Supposing that the irritation of lactation might have some effect on her general health I directed her to wean her child which she did. Gave her alterative doses of calomel combined with ipecac, followed by laxatives of rhubarb, sub. carb. soda, with a wash of nitrate of silver. Continued this treatment for a short time, during which her mouth was much improved, and also her general health.

In about a month after, however, her gums and mouth became suddenly worse, with much prostration of her whole system; a number of petechial spots made their appearance about the same time on her lower extremities. The fungous excresences extended around both jaws, as represented by the cut, but was much worse on the right side of the under jaw; they rose above the chewing surface of the teeth, so that she could not close them in front by three-eighths of an inch, and any attempt to do so was attended with severe pain; the gum opposite the second incisor on the left side of the upper jaw was the only place, on either jaw, that remained healthy. Breath very offensive; teeth all sound to appearance except the dentes sapientiæ of the left side of the upper jaw, but incrusted with tartar.

*Treatment.*—Commenced with a brisk cathartic of calomel and oil; after which I ordered the arsenite of potash in doses of four drops, to be gradually increased to eight; after the use of this article for two weeks it began to show its specific effects; at the same time the patient was directed to use a wash for the mouth of a solution of creosote, thirty drops to the ounce of water. But very little improvement in the gums could be noticed; general health better; petechial spots disappearing.

Arsenite of potash continued after a suspension of four days. I then incised the fungous gums freely, and on the right side of the under jaw clipped off some of them, which caused considerable hemorrhage; I then commenced with a strong solution of nitric acid as a wash for the mouth:

℞ Nitric acid, ʒiij.

Water, ℥iij.

M.

I visited her every third or fourth day, and at each time incised the gums freely, and when the fungous growth protruded cut it off.

From the time I commenced the incisions and the use of the acid, the fungus began to recede. As soon as it had declined sufficiently, I extracted the second molar tooth, and as well as I could, cleansed the balance from tartar.

After a continuation of the above treatment for two months the general health was much improved; the mouth and gums nearly well; the place from which the tooth was taken well; I advised her to have some more of them taken out, but she declined.

Sept. 17th. Saw Mrs. McC., she expresses herself quite well except yet rather weak; her gums are entirely well; teeth not injured by the acid. She is now able to attend to her domestic employment.

The plate accompanying this case was engraved by Mr. H. Anderson of this place, and is a faithful representation of the patient's mouth at its worst stage.

September, 1845.

## Figures and Tables

**Figure f1:**